# Supported Decision-Making in Persons With Dementia: Development of an Enhanced Consent Procedure for Lumbar Puncture

**DOI:** 10.3389/fpsyt.2021.780276

**Published:** 2021-11-16

**Authors:** Theresa S. Wied, Julia Haberstroh, Jakov Gather, Tarik Karakaya, Frank Oswald, Mishal Qubad, Matthé Scholten, Jochen Vollmann, Johannes Pantel, The ENSURE Consortium, Julia Haberstroh

**Affiliations:** ^1^Geriatric Medicine, Institute of General Practice, Goethe University Frankfurt, Frankfurt, Germany; ^2^Psychological Aging Research, Institute of Psychology, University of Siegen, Siegen, Germany; ^3^Institute for Medical Ethics and History of Medicine, Ruhr University Bochum, Bochum, Germany; ^4^Department of Psychiatry, Psychotherapy and Preventive Medicine, LWL University Hospital, Ruhr University Bochum, Bochum, Germany; ^5^Department of Psychiatry, Psychosomatic Medicine and Psychotherapy, University Hospital, Goethe University, Frankfurt, Germany; ^6^Interdisciplinary Ageing Research, Faculty of Educational Sciences, Goethe University Frankfurt, Frankfurt, Germany

**Keywords:** dementia, supported decision-making, informed consent, autonomy, lumbar puncture

## Abstract

The right to make autonomous decisions is enshrined in law. However, the question how persons with cognitive deficits can be enabled to make autonomous decisions has not been satisfactorily addressed. In particular, the concept of supported decision-making and its implementation into practice has been poorly explored for persons with dementia (PwD).This article describes the empirical development and implementation of support tools to enhance informed consent processes (so called enhanced consent procedures/ECP) for PwD on whether to undergo lumbar puncture. In the end of the process of pilot testing and further development of the tools, the following tools were defined: (1) Standardized Interview Structure, (2) Elaborated Plain Language, (3) Ambience and Room Design, (4) Keyword Lists, (5) Priority Cards, (6) Visualization, and (7) Simplified Written Informed Consent (Patient Information), as well as the general attitude (8) Person-Centered Attitude of the facilitator. As the development, implementation and evaluation of ECP tools is one objective of the transnational ENSURE project, we also include an overview of future empirical procedures. So far, our findings can serve as a selection of possibilities to support PwD in decision-making and help practitioners achieve an appropriate balance between the autonomy and protection of PwD in complex decision-making situation. Future studies should address the question if the proposed set of tools is effective to enhance informed consent processes in PwD.

## Introduction

Individual autonomy encompasses self-determined decision-making in medical and research contexts. The UN Convention on the Rights of Persons with Disabilities (UN-CRPD) is a human rights treaty that recognizes persons with disabilities, such as persons with dementia (PwD), as persons before the law with legal capacity and obliges state parties to support their ability to make decisions with legal effect (1). Similarly, the International Guidelines for Heath-related Research Involving Humans of the Council for International Organizations of Medical Sciences state that “adequate time and resources must be provided for informed-consent procedures” and that “researchers should use evidence-based methods for imparting information to ensure comprehension” (2).

The British *National Institute for Health and Care Excellence* (3) provides general recommendations on how to involve PwD in decision-making and how to provide information adequately (https://www.nice.org.uk/guidance/ng97). However, supported decision-making (SDM) in PwD requires further research. Its implementation in practice is rudimentary and the conceptual and theoretical framework vague. A systematic review of SDM in PwD (4) shows that it is–if at all–mostly applied in care and everyday-life contexts, but plays little role in treatment and research decisions.

In order to make treatment and research decisions, PwD need to participate in an informed consent (IC) process, which requires that: (1) a competent person (2) makes a free choice (3) following adequate information disclosure (5). Information disclosure is part of the IC process into which it would be possible to integrate SDM. Such approaches are called enhanced consent procedures (ECP) (6).

The “combined SDM model” (7), we adopt here combines decision-support with competence assessment. Decision-support has three possible outcomes: (1) Decision-making capacity (DMC) of PwD improves sufficiently to make an informed decision, and substitute decision-making becomes unnecessary; (2) DMC improves but decision-support was insufficient or inadequate and must be modified and provided again or; (3) DMC does not improve despite decision-support, and the PwD remains unable to make an informed decision.

The assessment of whether a person is competent is based on a concept of mental capacity that implies cognitive functioning. Grisso and Applebaum (5) defined four functional abilities: the ability to understand information, to appreciate its relevance, to reason it, and the ability to express a choice. Based on this concept they developed and validated a widely used instrument to assess mental capacity, the MacArthur Competence Tool (MacCAT) (5). Nowadays the MacCAT serves as a “gold standard” for the assessment of patients decisional capacity.

Beyond ethico-legal requirements, the highly internalized IC process is characterized by different attributes, such as the transfer of a huge amount of information (8), the use of technical medical terms, the separation of roles into experts and laypersons, and a potentially resulting imbalance of power (9, 10).

Further research is needed on how to implement SDM in PwD, how to enhance IC processes for PwD in treatment and research decisions, and how to provide adequate decision-support.

This study is part of the broad transnational ENSURE project (Enhancing the Informed Consent Process: Supported decision-making and capacity assessment in clinical dementia research). The development, implementation, and evaluation of tools to enhance the IC process for PwD is one of the transnational project partner's four objectives.

First steps toward achieving this objective are to identify appropriate support measures and to examine their potential for transfer to different decision-making situations. We choose the decision for lumbar puncture because (1) lumbar puncture constitutes a medical procedure whose IC process is precisely defined, (2) it is an important part of diagnostic work-up in certain cases (3), and (3) many clinical trials conducted with PwD include lumbar puncture. Against this background, the aim of this article is to outline the empirical development of decision-support, so called tools, to enhance this process.

## Method

### Development of Tools

Based on five defined criteria, those more general support measures were selected from the systematic literature review (4) that should be considered in the tools. The following five criteria were defined: (1) multiple answers, (2) compliance with published recommendations by experts on the capacity to give consent, (3) compliance with the (clinical) experience of the ENSURE team's experts, (4) effectiveness and (5) practicability. The support measures selected with the help of the named criteria, were ordered and bundled afterwards on the starting point of complexity reduction. The process of the development of tools as well as the five criteria for selecting support measures identified in the systematic literature review (4) are described in detail elsewhere (11).

### Pilot Testing and Further Development of Tools

The defined tools and their application instructions were piloted and further developed in two processes. On the one hand, the first drafts of tools were implemented in real clinical IC procedures for a lumbar puncture from January 2018 and optimized together with the applying physician. Therefore, ethical approval was obtained from the local ethics committee of the University Hospital Frankfurt. On the other hand, the further development included an iterative process involving discussions among the members of the Ensure Consortium (ethicists, legal experts, nursing scientists, physicians, psychologists). This process was used to revise content and structure of the tools and to reflect upon ethical and practical challenges until consensus regarding appropriateness was reached. Issues like overburdening, overcompensation, sidestepping memory, interpersonal leverage, oversimplification, issue framing, and criteria for allocation of the support were discussed within the consortium.

### Recruitment, Participants, and Setting

Together with our practice partners we recruited persons with suspected dementia that had been admitted to a psychogeriatric ward because of subjectively experienced cognitive impairment. Lumbar punctures had been recommended to the patients by their physicians for diagnostic work-up. Thus, we introduced the newly developed tools in an IC process that would have occurred anyway. In few cases the LP war performed immediately after the ECT, usually within 1 to 2 days after it.

Fourteen persons with suspected dementia participated in the ECPs, 11 of whom had been diagnosed with dementia or mild cognitive impairment (MCI) at the time of discharge from hospital. One ECP had to be terminated due to strong emotional stress of the participant. Of the 14 ECP conducted, 10 people with dementia or MCI (7 women, 3 men) could be finally included in the study. The participants were on average 67.5 years old (range 54–78). Two of them were diagnosed with Alzheimer's at the time of hospital discharge, four with unspecified dementia, and four with MCI. The participant's mean Mini Mental State Examination (MMSE) score was 24.3 (range 21–27).

### Training of the Physician

The physician who was in charge of the lumbar puncture was trained to use the tools, and the entire ECP was carried out on a hypothetical case. One researcher (TW) attended all ECPs and assisted the physician with the implementation of the tools. A thirteen-page moderation-plan was written for conducting the ECPs.

### Optimization of the Tools

Following the first implementation, we successively adjusted and optimized the applied tools regarding their feasibility. The attending researcher (TW) discussed each conducted ECP with the physician. They reviewed observations made during the ECP and jointly identified optimization potential. Subsequently the research team refined the tools again. The adjustments made are displayed in Table 1. For example, we initially used one keyword list containing bulleted keywords to describe the three information sequences, understanding the disease, understanding the treatment, and understanding the risks and benefits (Mac-CAT). The first interviews revealed that our participants were overwhelmed by the variety of keywords and were constantly searching for the related keywords on the list. We therefore decided to employ one list for each of the three information sequences.

**Table 1:** Adjustments to applied support tools.

**Tool / Attitude**	**Adjustments**
Person-centered attitude	•The person-centered attitude of the facilitator was initially handled as an independent tool before it was decided to define it as a basic attitude that must be practiced as a basis for the application of other tools
	•After telling the patient about a suspected diagnosis of dementia, we took a break. We emphasized that dementia was suspected (especially at the initial diagnosis)
Standardized interview structure	•We included standardized breaks after each information sequence
	•If a participant wanted something repeated, we instructed the physician to repeat the whole information sequence (Mac-CAT)
	•We instructed the physician to assist in reproducing information if necessary, e.g., by naming keywords from the required responses (Mac-CAT)
	•We added missing information
Elaborated Plain language	•Sentences and wording were continually simplified, e.g.
	“We can use the needle to withdraw a few milliliters of spinal fluid.”
	“We can take a little spinal fluid *via* the needle.”
Ambience and room design	•We chose another room in preference to the doctor's room (room for occupational therapy)
	•Before participants were brought in, we prepared the room (tidiness, fresh air, heating)
Keyword lists	•We divided up the keywords and employed one list for each of the three information sequences
	•The keyword lists were taken back after each check of understanding so that participants had a maximum of one list in front of them
Priority cards	•We instructed the physician to ask our participants to explain the significance of only the “important” cards, rather than all of them
Visualization	•We changed the pictogram
	The spine was drawn more realistically and transparently
	Person on the picture was depicted as more ageless
Enhanced written Consent form (patient) information	•Modified according to the new pictogram

*Note: Mac-CAT, Mac Arthur Competence Assessment Tool; e.g., for example*.

## Results

In the end of the process of pilot testing and further development of the tool, the following tools were defined: (1) Standardized Interview Structure, (2) Elaborated Plain Language, (3) Ambience and Room Design, (4) Keyword Lists, (5) Priority Cards, (6) Visualization, and (7) Simplified Written Informed Consent (Patient Information), as well as the general attitude (8) Person-Centered Attitude of the facilitator. Instructions for use have been formulated for each tool.

### General Attitude: Person-Centered Attitude of the Facilitator

Current research into decision-making needs and demands of PwD shows that the facilitator's attitude should be person-centered (4). This means providing subtle support and considering PwD as equal partners in the decision-making process, rather than taking over decision-making (12). Even if it seems self-evident, person-centeredness must be borne in mind and practiced, and we instructed physicians to have such an attitude during the IC process. In this respect, relationship aspects of communication need to be considered (13). In our written schedule, we gave such advice as:

- Invite participants, welcome them using their names.
- Introduce yourself with first, last name and function, if not yet known.
- Offer participants a chair and something to drink.
- Take a seat yourself.
- Establish and maintain eye contact.
- Provide time for questions, allow breaks if necessary.
- Clarify that you are available to take further questions after the ECP; say goodbye.

Prior to implementation, advice and recommendations were discussed with the physician.

### Tool 1: Standardized Interview Structure

When obtaining IC, a structured approach and an open interview-style appeared crucial. To structure the IC process and reduce its complexity, information could therefore be presented in shorter segments (14), and the understanding of the PwD verified (14–16). In dementia care networks, PwD recognized the need for a decision more easily when others raised and introduced topics slowly, and clearly initiated the decision-making process (17). Besides a clear structure, the interview-style should encourage dialog and enable PwD to express themselves (16). Furthermore, decision-making should consider the pace of PwD, i.e., allow extra time or slow down the discussion where necessary (12, 14, 16–18).

To provide a supportive structure, we decided to use the MacArthur Competence Tool (MacCAT-T) to obtain IC and assess competency to consent to treatment. The Mac-CAT interview is performed in a standardized way by providing fixed sequences of information and then asking questions, inter alia, to verify understanding (5). Furthermore, the physician raises the decision-making topic and introduces it slowly, and it is clear when decision-making begins. We instructed the physician to ask the PwD for questions after each information sequence, and breaks were offered frequently.

To explain the IC procedure and clarify the structure of the ECP for the physician, we designed a detailed plan of the IC procedure (written schedule) in accordance with the MacCAT-T. It included an exemplary script and the timely application of further tools.

### Tool 2: Elaborated Plain Language

The consideration of language aspects (15, 16, 18) may help PwD understand and minimize verbal demands. Schatz et al. (19) describe ways to improve the presentation of information in IC processes. By applying the first rule of so-called plain language to “use language the audience knows and feels comfortable with” (20) and referring only to the two positively evaluated characteristics of the otherwise criticized Elderspeak [e.g., (21)], we introduced elaborated plain language (EPL) (19). It is a clear and simple language with four main attributes. The EPL was applied throughout the ECP.

(1) We focused on the reduction of syntactical complexity, which means shortening sentences and using fewer subordinate clauses. We used only one subordinate clause per main clause and avoided convoluted sentences.
(2) We introduced semantic elaborations, which refer to the provision of further information (expansions) and an iteration of keywords by allowing them to “move” from sentence to sentence: “*There are also* side effects *in the investigation. The most common* side effect *is* headache*, which can occur up to five days after the withdrawal of spinal fluid. The* headaches *improve when you lie down and drink a lot.”*
(3) We limited the vocabulary, which means we avoided technical terms, e.g., “image of the brain” rather than “CT scan.”
(4) We focused on neutral prosody that includes the avoidance of a slow speaking rate, high pitch, and short sentences (21).

### Tool 3: Ambience and Room Design

In order to facilitate decision-making and clarify choices (15), describe how caregivers simplify the decision-making environment by removing unimportant objects and keeping things tidy. Under “keeping it simple” (18) write that care staff recommend avoiding distracting and noisy environments, e.g., with too many attendees. Moye et al. (14) also recommend minimizing background noise.

To avoid sidetracking stimuli, we chose a separate room for the IC process in preference to the doctor's office. The room, which is usually used for occupational therapy, has no telephone connection or computer access, and is located at the end of the corridor of the ward. We asked the physician to leave her phones outside during the IC process and told other health care practitioners on the ward not to disturb us for the next 30 min. In addition we placed a “please do not disturb” sign on the door and closed it. Apart from the patient (and sometimes a relative), only two persons attended the process (researcher, physician). The selected room has a large window providing natural light. The table the attending persons were sitting at was kept tidy.

### Tool 4: Keyword Lists

While Smebye et al.(15) describe compensating for the failing memory of PwD by using aids and props, Haberstroh et al. (22) recommend memory-based strategies that reduce verbal memory loads and facilitate verbal retrieval. PwD understanding improved in a study by Rubright et al. (23), who enhanced the IC process with a memory and organizational aid that introduced, for example, summarized key elements. Moye et al.(14) recommend summarizing “[…] key aspects of information, such as reviewing key risks and benefits of each treatment, prior to asking the patient for treatment preference.”

To achieve this, we applied additional lists with bulleted key information. We summarized the most important information by using easy-to-read language, and wrote the key points on a number of lists, each containing a manageable amount of information. The keyword lists were provided in addition to verbal information, thus utilizing iteration by presenting information repeatedly (spoken and written).

We instructed the physician to hand over each list after the information had been provided verbally, ask the PwD to summarize what he or she had been told, and then to take the list away before starting the next information sequence.

### Tool 5: Priority Cards

To help PwD draw conclusions, compare the influence of lumbar puncture with its alternatives, and gauge its risks and benefits with respect to their situation and everyday life, we developed so-called priority cards. The communication framework Talking Mats, which aims to facilitate communication in the decision-making process (24), inspired this process.

Priority Cards enable PwD to visualize the risks and benefits of treatment on a single card containing a visual scale (important, not important). We asked PwD to use the scale to organize each card in accordance with their priorities. Furthermore, they had the option to express their own views on the treatment. During the ECP, the attending researcher wrote such reasons down on a blank card. After organizing the cards, we asked our participants to explain why the “important” cards were significant to them, and what effects the risks and benefits would have, regardless of whether they provide their consent.

### Tool 6: Visualization

Featherstonehaugh et al. (18) describe how care staff tried to facilitate PwD's decision-making by “showing” alternatives, or using visual representations, such as pictures of a menu. Such non-verbal content aspects could be considered, to support the understanding of PwD in more complex situations by minimizing verbal memory load (13). A clinical strategy to maximize decisional capacity involves the use of such cues as pictures and diagrams (14).

**Figure 1:**
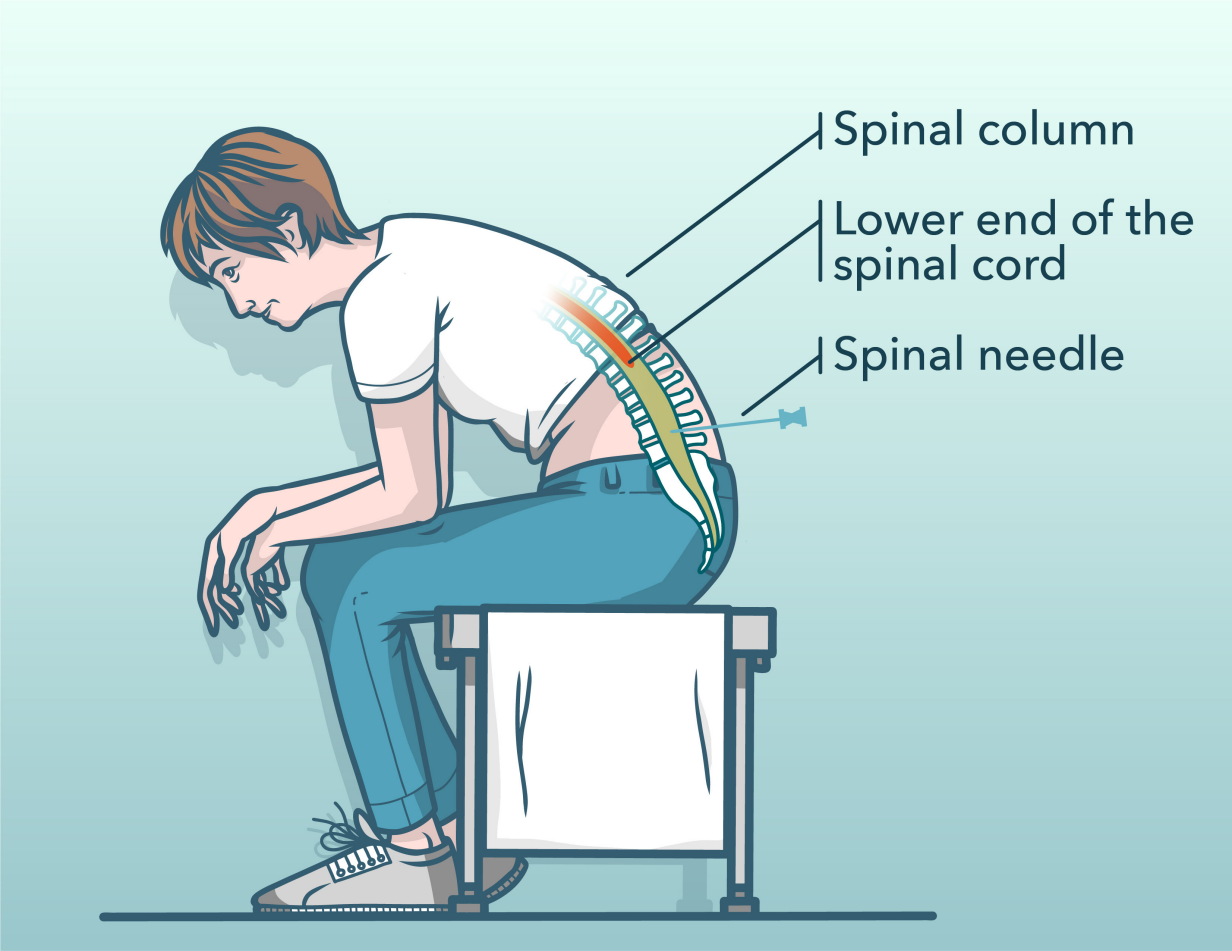
Illustrating pictogram of the diagnostic procedure used for the enhanced consent procedure.

We developed a pictogram to help PwD understand the treatment, and more specifically, the puncture site of the needle and the posture during treatment (see Figure 1). The aim of the pictogram is to simplify and iterate verbal information through visualization: “*In a spinal tap, fluid is taken from the spinal canal. For this purpose, you will be stung below the spinal cord with a needle (pointing gesture toward back). […] For the withdrawal of the spinal fluid, you need to sit (or lie) still for some time. […] (pointing gesture toward pictogram).”*

Directing the attention of PwD to important aspects (6, 23) could reduce the complexity of the process and indicates to PwD what is particularly significant. Hence, we introduced pointing gestures within the ECP, e.g., “*We also record the conversation (pointing gesture toward recording device).”*

### Tool 7: Enhanced Written Consent Form (Patient Information)

We prepared our own enhanced written consent form (patient information) in preference to the hospital's to allow more time to decide (18), to avoid coercing the PwD (17), and to enable decisions to be revoked or modified (16).

We wanted to give PwD the opportunity to read received verbal information again (iteration) and to permit them to review their preliminary decisions, thus facilitating ongoing consent. Re-reading the information in a more relaxed atmosphere, possibly with a trusted person, may trigger further questions, which could then be clarified in another discussion with the physician. Signing the consent form was mostly postponed until later.

Written patient information was kept short (two pages) and delivered information in the same sequence as the verbal presentation. It includes visualization, hence the developed pictogram (see Tool 6). We simplified the enhanced written consent form information by using elaborated plain language^1^

## Discussion

This article describes the systematic development of support-tools for an ECP for lumbar puncture treatment for PwD. By involving an interdisciplinary transnational expert group, ethical, legal, and practical concerns were all considered equally. Our practical experience increased during each ECP, e.g., how to elicit the opinions of PwD, how often to offer breaks, how manageable the number of keywords on a single keyword list is, and how to apply elaborated plain language.

First observations of the researcher who assisted the implementation process (TW) support the assumption that many tools could simplify the IC or at least did not cause negative effects on the participants. For example, the visualization *via* pictogram “explained everything” to one participant, it appeared “a bit scary” to another. While the standardized interview structure enabled dialog in some cases, in other cases, the questions were perceived as “intensive” and participants seemed to feel tested by the physician. A deeper analysis of the interview and observation data will provide important insights. The applying physician evaluated the ECP as useful and intend to maintain some tools in future ICs.

The next steps of our project included an empirical evaluation of the tools within a small sample of PwD which is described elsewhere (11). The study provides initial indications that some participants felt supported by individual tools and that the targeted reduction in complexity in the informational dimension was successful in some cases. This enabled us to include their views as users of the support-tools and the ECP. A follow up study with the inclusion of patient's perspectives and assessment of patient's satisfaction should be performed in a larger cohort.

In this step the tools were not yet been evaluated in terms of their effectiveness (e.g., improved understanding, appreciation, reasoning, or overall score of mental capacity) but rather in terms of their feasibility, acceptability and appropriateness for the affected PwD. This evaluation was based on problem-centered interviews with every ECP participant. An additional small study was conducted, to investigate dementia researchers view on the developed tools (25). In brief, we performed an online survey with 19 dementia researchers from Germany and Portugal and evaluated the tools in terms of 4 implementation criteria. Overall, all researchers had a very positive attitude toward the support tools, whereby the tools person-centered attitude of the researcher and elaborated plain language were the most highly rated of the eight tools. Our findings also indicated that familiar support tools were assessed more favorably than those that were previously unknown. This demonstrated that the participating dementia researchers were open to the use of decision support measures in PwD and were willing to apply the support tools in practice.

We recognize that every PwD must be considered and treated as an individual with his or her own views, needs, abilities, and impairments. Therefore, we do not recommend applying all eight tools in standard form, but rather suggest selecting tools according to the individual needs of the single PwD and the resources (e.g., time and room availability) and abilities (e.g., qualifications) of the practitioner. Our findings can serve as a selection of possibilities to support PwD in decision-making and help practitioners achieve an appropriate balance between the autonomy and protection of PwD in complex decision-making.

Future studies should address the question if the proposed set of tools is not only feasible but also effective to enhance informed consent processes in PwD.

## Data Availability Statement

The raw data supporting the conclusions of this article will be made available by the authors, without undue reservation.

## Ethics Statement

The studies involving human participants were reviewed and approved by Ethikkommission des Fachbereichs Medizin der Goethe Universität Frankfurt, Deutschland. The patients/participants provided their written informed consent to participate in this study.

## Ensure Project: Enhancing the Informed Consent Process

The ENSURE Consortium was a transnational project team of interdisciplinary researchers: Goethe-Universität Frankfurt (Julia Haberstroh, Frank Oswald, Johannes Pantel, Theresa Wied), Ruhr-Universität Bochum (Jakov Gather, Matthé Scholten, Jochen Vollmann), Universidade da Coruña (Nathalia Álvarez Lata, José-Antonio Seoane), Universidade Católica Portuguesa (Ana Sofia Carvalho, Pablo Hernández Marrero).

## Author Contributions

TW and JH: substantial contributions to the conception and design of the work. TW, JH, JP, JG, MS, JV, and FO: substantial contribution to the analysis or interpretation of data for the work. TW and TK: substantial contribution to the acquisition of data. TW, JP, and JH: drafting the work or revising it critically for important intellectual content. TW, JH, JG, JQ, TK, FO, MQ, MS, JV, and JP: provide approval for publication of the content and agree to be accountable for all aspects of the work in ensuring that questions related to the accuracy or integrity of any part of the work are appropriately investigated and resolved. All authors contributed to the article and approved the submitted version.

## Funding

This work was funded by the Network of European Funding for Neuroscience Research (ERA-NET NEURON), the German Federal Ministry of Education and Research (Grant No. 01GP1623A), and the Volkswagen Foundation.

## Conflict of Interest

The authors declare that the research was conducted in the absence of any commercial or financial relationships that could be construed as a potential conflict of interest.

## Publisher's Note

All claims expressed in this article are solely those of the authors and do not necessarily represent those of their affiliated organizations, or those of the publisher, the editors and the reviewers. Any product that may be evaluated in this article, or claim that may be made by its manufacturer, is not guaranteed or endorsed by the publisher.

## Footnotes

^1^We are ready to share the latest version of the written patient information and other relevant material with interested researchers and clinicians upon request.
